# Pooling overdispersed binomial data to estimate event rate

**DOI:** 10.1186/1471-2288-8-58

**Published:** 2008-08-19

**Authors:** Yinong Young-Xu, K Arnold Chan

**Affiliations:** 1 EpiPatterns, Haverhill, NH, USA; 2 Department of Epidemiology, Harvard School of Public Health, Boston, MA, USA; 3 i3 Drug Safety, Waltham, MA, USA

## Abstract

**Background:**

The beta-binomial model is one of the methods that can be used to validly combine event rates from overdispersed binomial data. Our objective is to provide a full description of this method and to update and broaden its applications in clinical and public health research.

**Methods:**

We describe the statistical theories behind the beta-binomial model and the associated estimation methods. We supply information about statistical software that can provide beta-binomial estimations. Using a published example, we illustrate the application of the beta-binomial model when pooling overdispersed binomial data.

**Results:**

In an example regarding the safety of oral antifungal treatments, we had 41 treatment arms with event rates varying from 0% to 13.89%. Using the beta-binomial model, we obtained a summary event rate of 3.44% with a standard error of 0.59%. The parameters of the beta-binomial model took the values of 1.24 for alpha and 34.73 for beta.

**Conclusion:**

The beta-binomial model can provide a robust estimate for the summary event rate by pooling overdispersed binomial data from different studies. The explanation of the method and the demonstration of its applications should help researchers incorporate the beta-binomial method as they aggregate probabilities of events from heterogeneous studies.

## Background

In clinical research and public health, it is frequently necessary to combine findings from multiple interventional or observational studies in order to address important safety and efficacy questions. A single study rarely provides a definitive answer because of limited sample size and the specific attributes of particular study populations. The challenges of combining data from heterogeneous studies are well described in the meta-analysis literature. In the majority of meta-analysis reports, the outcome of interest is a comparative risk estimate such as the odds ratio, relative risk, or risk difference [1]. Absolute risks, however, such as the proportion of clinical events among a cohort of patients or the response rate among patients receiving a certain treatment regimen, are important measures for helping to guide clinical and public health decisions. In the correct epidemiology and statistical terminology, these so-called rates are really proportions, but we will treat rates and proportions as equivalent in this paper as this term is commonly used in medical product safety research. Relevant methods to pool the absolute risks are especially important in safety evaluation of medical products as the risks for serious adverse outcomes are often rare, and precise estimates of the probability of these outcomes are crucial in the risk-benefit evaluation.

In this report we describe the implementation of the beta-binomial method to pool the absolute risks from overdispersed data. This method estimates a summary probability of adverse events and is applicable in medical product safety evaluation as it takes into account the heterogeneity of studies. The application of the beta-binomial method in drug safety settings was previously described by Chuang-Stein in 1993 [2]. Here we aim to provide a detailed description of the method and to update and broaden its applications.

The general setting is that of a clinical trial or cohort study of a specific exposure, such as: drug A with a sample size of *n *resulted in *x *number of adverse events (e.g. liver injury). Within each individual study the probability of encountering *x *number of adverse events out of a sample size of *n *is characterized by the binomial distribution. To summarize multiple studies of the same exposure, we need to account for their heterogeneity of the studies, for they could differ in their sample sizes, clinical settings, investigators, protocols, and prevalence of comorbidity among study subjects. The assumption of one binomial distribution that can describe the proportions of adverse event from all the studies is not always valid. Numerous factors, including ethnic difference, disease severity, comorbid conditions, and concomitant medications can contribute to the variation of the probability of interest, thus requiring additional assumptions beyond the binomial model. This phenomenon is often referred to as overdispersion [3,4]. Ignoring overdispersion when pooling overdispersed data that are binomial in nature could result in erroneous estimates of the probability of interest and its confidence interval.

In the clinical trial literature, Chuang-Stein [2] proposed using the beta-binomial model to combine binomial event rates across multiple studies in an article titled "An application of the beta-binomial model to combine and monitor medical event rates in clinical trials." Despite its sound statistical basis, this method has not been widely used in clinical and public health research articles during the years since its publication. Meanwhile, the application of the beta-binomial model in other fields is becoming more prevalent as it has been applied in fields as distant as sensory analysis [5] and computational linguistics [6]. We utilized this method to estimate the risk of liver toxicity among users of oral antifungal treatments [7] and believe that it can be used more widely to help address similar questions. In the rest of this article we describe the statistical assumptions for the beta-binomial model, the process of estimating the probability of interest, methods to test for over-dispersion, and an example of its application.

## Methods

### The Beta-Binomial distribution

Both Chuang-Stein [2] and Ennis [5] provide excellent references for those who are interested in the history of the beta-binomial model. Recall the definition of the binomial distribution:

(1) $$\begin{array}{cc} \text{Prob}(X=x)=\left(\begin{array}{l} n\\ x \end{array}\right)\;p^{x}{\left(1-p\right)}^{n-x}, & 0\leq p\leq 1 \end{array}$$

where *x *is the number of successes in a sequence of *n *independent success/failure experiments, each of which has probability *p *for success.

**Figure 1:**
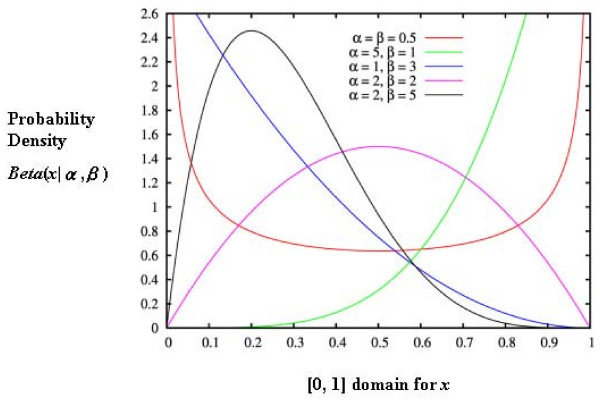
Variety of shapes for beta distributions.

Let probability *p *follow a beta distribution (*p*|*α, β*), then

(2) $$\text{Beta}(p|\alpha ,\beta )=\frac{\Gamma (\alpha +\beta )}{\Gamma (\alpha )\Gamma (\beta )}p^{\alpha -1}{\left(1-p\right)}^{\beta -1}$$

where *Γ *is the gamma function over the domain [0, 1]; *α *and *β *are two positive parameters. The beta distribution was selected in the past because of its flexibility (capable of a wide range of shapes, see Figure 1) and its ability to provide good approximations. As Skellam [8] stated as early as 1948, "in practice we could, at least in most cases, take this form of distribution as a convenient approximation." As a result, we arrive at a combination of the binomial distribution with a beta density function:

(3) $$\text{Prob}(X=x)=\left(\begin{array}{l} n\\ x \end{array}\right)\frac{\Gamma (\alpha +\beta )\Gamma (\alpha +x)\Gamma (\beta +n-x)}{\Gamma (\alpha )\Gamma (\beta )\Gamma (\alpha +\beta +n)}$$

where *x *takes on the values 0, 1, 2... *n*, and *α *and *β *are positive. Note in equation (3) that *n *is the total number of study subjects, and *x *is the total number of subjects with a certain adverse event, although what most investigators are interested in is the proportion *p *that varies between 0 and 1 and has the appearance of a continuous distribution.

So let *p*_*i *_= *x*_*i*_/*n*_*i*_, *i *= 1,2, ... *k*, where *i *indexes the different studies, *x*_*i *_is the number of events in the *i*th study and *n*_*i *_is the sample size of the study. To reiterate, within the context of multiple studies where each study with sample size *n*_*i *_and binomial probability *p*_*i *_(e.g. for adverse events), one binomial distribution cannot adequately describe the additional variation when *p*_*i *_varies and thus the data are fitted with a beta distribution with parameters (*α, β*), with *α *> 0 and *β *> 0. Let *μ = α/(α+β), θ = *1/*(α+β)*, where *μ *is the mean event rate (i.e., the expected value of a variable binomial parameter *p*) and *θ *is a measure of the variation in *p*. In short, we have constructed a two-stage model:

*X*_*i *_| *p*_*i *_~*Bin*(*n*_*i*_, *p*_*i*_)

*p*_*i*_~*Beta *(*μ, θ*),*i.i.d*

The mean and variance of *X *are *nμ *and *nμ(*1-*μ){θ/(*1+*θ)} *[9]. One can view the term {*θ/(*1+*θ)} *as a multiplier of the binomial variance. In other words, it models the overdispersion. Some authors (e.g. Kleinman [10]) prefer the term *γ *where *γ = θ/(*1+*θ) = *1/*(α+β+1)*. Then the variance is *nμ(*1 *- μ) γ*. In essence, one can derive the same information from *θ *and *γ *about the beta-binomial distribution, so it is beneficial to know both and employ whichever is more convenient for computation.

### Estimation of Parameters

Two main methods, one involving moments and the other involving maximum likelihood, are often used to estimate the parameters *μ *and *θ*.

### The Moment Estimates Method

In terms of actual data observed from different studies, let *p*_*i *_= *x*_*i*_/*n*_*i*_, *i *= 1,2, ... *k*, where *i *indexes the different studies, *x*_*i *_is the number of events in the *i*th study and *n*_*i *_is the sample size of the study. The *n*_*i*_*'s *here are almost always unequal in clinical studies.

Let

(4) $$\begin{array}{ccc} \hat{p}=\frac{\sum_{1}^{k}w_{i}{\hat{p}}_{i}}{w},w=\sum_{1}^{k}w_{i} & \text{and} & w_{i}=\frac{n_{i}}{1+\gamma (n_{i}-1)} \end{array}$$

where {*w*_*i*_} represents a set of weights and *w *is the sum of all the weights [10].

Let also $S=\sum_{i=1}^{k}w_{i}(p_{i}-\hat{p}{)}^{2}$

then the moment estimates of *μ *and *γ *are:

$\hat{\mu }=\hat{p}$ and

(5) $$\hat{\gamma }=\frac{S-\hat{p}\hat{q}\left[\sum \frac{w_{i}}{n_{i}}(1-\frac{w_{i}}{w})\right]}{\hat{p}\hat{q}\left[\sum wi(1-\frac{w_{i}}{w})-\sum \frac{w_{i}}{n_{i}}(1-\frac{w_{i}}{w})\right]}$$

where $\hat{q}=1-\hat{p}$. To derive *θ*, we can simply perform the following conversion:

*θ *= *γ*/(1 - *γ*)

Providing the proper set of weights is challenging because {*w*_*i*_} is a function of the unknown parameter *γ*. Kleinman [10] first offered an empirical weighting procedure and suggested to set *w*_*i *_= *n*_*i *_or *w*_*i *_= 1 to obtain an initial approximation of estimates of *μ *and *γ *using equation (4). Using this estimation of *γ *to compute {*w*_*i*_}, one then can use these "empirical" weights to arrive at a new estimate of *μ*. In cases where *γ *estimates are negative, they are to be set to zero. Chuang-Stein [2] proposed an improvement on Kleinman's procedure by suggesting that the iteration be carried further until the differences between two consecutive sets of estimates for *μ *and *γ *are both smaller than some predetermined value. The example that was given in the paper [2] was 10^-6^.

Notations are simpler in cases where all *n*_*i*_*'s *are equal, then

$$\begin{array}{ccc} \hat{p}=\left(\sum_{i=1}^{k}p_{i}\right)/k & \text{and} & S=\sum_{i=1}^{k}(p_{i}-\hat{p}{)}^{2} \end{array}$$

The moment estimates of *μ *and *γ *are

$\hat{\mu }=\hat{p}$ and

(6) $$\hat{\gamma }=\frac{nS}{\hat{p}(1-\hat{p})k(n-1)}-\frac{1}{n-1}.$$

### The Maximum Likelihood Estimates Method

As is written above, let *p*_*i *_= *x*_*i*_/*n*_*i*_, *i *= 1,2, ... *k*, where *i *indexes the different studies, *x*_*i *_is the number of events in the *i*th study and *n*_*i *_is the sample size of the study. The maximum likelihood (ML) function involving *α *and *β *can be written as

$$L(\alpha ,\beta )=\prod_{x=0}^{k}\left(\begin{array}{l} n\\ x \end{array}\right)\frac{B(\alpha +x,\beta +n-x)}{B(\alpha ,\beta )}$$

where $B(\alpha ,\beta )=\frac{\Gamma (\alpha )\Gamma (\beta )}{\Gamma (\alpha +\beta )}$ is the beta function of *α *and *β *and is used here to simplify equation (3). The log likelihood function is then

(7) $$c-\sum_{i=1}^{k}n_{i}\ln(B(\alpha ,\beta ))+\sum_{i=0}^{k}\ln(B(\alpha +x_{i},\beta +n_{i}-x_{i}))$$

where c is a constant. Next we will need to take the partial derivative of the log likelihood function with respect to *α *and *β*. The ML equations involving *α *and *β *are

(8) $$\begin{array}{l} 0=\frac{\partial \ln L}{\partial \alpha }=\sum_{i=1}^{k}\Delta_{1}(\alpha ,x_{i})-\sum_{i=1}^{k}\Delta_{1}(\alpha +\beta ,n_{i})\\ 0=\frac{\partial \ln L}{\partial \beta }=\sum_{i=1}^{k}\Delta_{1}(\beta ,n_{i}-x_{i})-\sum_{i=1}^{k}\Delta_{1}(\alpha +\beta ,n_{i}) \end{array}$$

where

$$\Delta_{1}(m,n)=\frac{1}{m+n-1}+\frac{1}{m+n-2}+...+\frac{1}{m}$$

The second derivatives of ln*L *are:

$$\frac{\partial^{2}\ln L}{\partial \alpha^{2}}=-\sum_{i=1}^{k}\Delta_{2}(\alpha ,x_{i})+\sum_{i=1}^{k}\Delta_{2}(\alpha +\beta ,n_{i})$$

$$\frac{\partial^{2}\ln L}{\partial \beta^{2}}=-\sum_{i=1}^{k}\Delta_{2}(\beta ,n_{i}-x_{i})+\sum_{i=1}^{k}\Delta_{2}(\alpha +\beta ,n_{i})$$

$$\frac{\partial^{2}\ln L}{\partial \alpha \partial \beta }=\sum_{i=1}^{k}\Delta_{2}(\alpha +\beta ,n_{i})$$

where

$$\Delta_{2}(m,n)=\frac{1}{{\left(m+n-1\right)}^{2}}+\frac{1}{{\left(m+n-2\right)}^{2}}+...+\frac{1}{m^{2}}$$

These second derivatives of the log likelihood function can be used to form the Hessian matrix which, in turn, can be used to derive the standard errors for the parameters. An example will be given in a following section. Most often *μ *is the main parameter of interest, and therefore we present a direct estimation of it rather than proceeding through *α *and *β*.

Define *f*_*x*_*(x) (x = 0,1,2, ..., k) *as the observed frequencies of events from *k *trials. Then the likelihood of beta-binomial can be also written as

$$L(\alpha ,\beta )=\prod_{x=0}^{k}{\left[P(x)\right]}^{f_{x}}.$$

Where *P(x) *has already been stated in (3). Let $S_{i}=\sum_{x=0}^{i}f_{x}(i=0,1,2,...,n)$ so that $S_{k}=\sum_{i=1}^{k}n_{i}$ is the total sample size of all the individual trials combined.

The log likelihood function in terms of *μ *and *θ *is

(9) $$c-S_{n}\sum_{i=1}^{n-1}\ln(1+i\theta )+\sum_{i=0}^{n-1}\left\{S_{n}-S_{i})\ln(\mu +i\theta )+S_{n-1-i}\ln(1-\mu +i\theta )\right\}$$

where *c *is a constant and the ML estimators of $\hat{\mu }$ and $\hat{\theta }$ are solutions of

$$0=\frac{\partial \ln L}{\partial \mu }|\hat{\mu },\hat{\theta }=\sum_{i=0}^{k-1}\left\{\frac{S_{k}-S_{i}}{\mu +i\theta }-\frac{S_{k-1-i}}{1-\mu +i\theta }\right\}$$

(10) $$0=\frac{\partial \ln L}{\partial \mu }|\hat{\mu },\hat{\theta }=\sum_{i=0}^{k-1}i\{\frac{S_{k}-S_{i}}{\mu +i\theta }+\frac{S_{k-1-i}}{1-\mu +i\theta }-\frac{S_{k-1-i}}{1+i\theta }\}$$

These equations can be solved iteratively using the Newton-Raphson method [11].

Again, the second partial derivatives of the log likelihood function can be used to form the Hessian matrix (H) at the ML solution

$$H(\hat{\mu },\hat{\theta })=\left[\begin{array}{cc} \frac{\partial^{2}\ln L}{\partial {\hat{\mu }}^{2}} & \frac{\partial^{2}\ln L}{\partial \hat{\mu }\partial \hat{\theta }}\\ \frac{\partial^{2}\ln L}{\partial \hat{\mu }\partial \hat{\theta }} & \frac{\partial^{2}\ln L}{\partial {\hat{\theta }}^{2}} \end{array}\right]$$

which, after being inverted, can be used to derive the covariance matrix and the standard errors for the parameters:

$$Cov(\hat{\mu },\hat{\theta })=\left[\begin{array}{cc} {\hat{\sigma }}_{\hat{\mu }}^{2} & \rho {\hat{\sigma }}_{\hat{\mu }}{\hat{\sigma }}_{\hat{\theta }}\\ \rho {\hat{\sigma }}_{\hat{\mu }}{\hat{\sigma }}_{\hat{\theta }} & {\hat{\sigma }}_{\hat{\theta }}^{2} \end{array}\right]$$

And the confidence intervals for $\hat{\mu }$ and $\hat{\theta }$ can be obtained by

(11) $$\hat{\mu }\pm Z_{1-\alpha /2}{\hat{\sigma }}_{\hat{\mu }}$$

(12) $$\hat{\theta }\pm Z_{1-\alpha /2}{\hat{\sigma }}_{\hat{\theta }}$$

where *Z*_1-*α*/2 _is the 1-*α*/2 percentile of a standard normal distribution function.

Once $\hat{\mu }$ and $\hat{\theta }$ are estimated, one can also derive $\hat{\alpha }$ and $\hat{\beta }$ from the relationships that *μ = α/(α+β), θ = 1/(α+β)*. It can easily be shown that the estimate of $\hat{\alpha }$ is $\hat{\mu }$/$\hat{\theta }$ and the estimate of $\hat{\beta }$ is (1 - $\hat{\mu }$)/$\hat{\theta }$. If we substitute these estimates for *α *and *β *in the beta-binomial model (3), then the cumulative distribution can be calculated.

As we have shown above, either method can be used to estimate the parameters of the beta-binomial distribution. Readers who are interested in more details should consult Griffiths [9] and Kleinman [10]. Researchers have implemented the maximum likelihood estimation (MLE) method in two popular commercial statistical software packages. In addition, free statistical software, such as R and WinBUGS, have methods for fitting the beta-binomial model, but they require some programming.

One of those two popular commercial statistical software packages is SAS (SAS Institute Inc., Cary, NC, USA). The macro BETABIN written by Ian Wakeling [12] is freely available. It borrows the existing SAS procedure NLMIXED to provide a maximum likelihood estimation of *μ *and *θ*. It provides not only the standard beta-binomial model, but also Brockhoff's [13] corrected beta-binomial model. Interested readers can also experiment directly with Proc NLMIXED to fit the beta-binomial model as others have done [14].

The other software is Stata (College Station, Texas). Guimarães provided the necessary computer commands for beta-binomial estimations using the Stata command xtnbreg with conditional maximum likelihood [15]. In addition, Guimarães emphasized the common knowledge that the beta-binomial distribution was a special case of the more general Dirichlet-multinomial (DM) distribution – with two parameters in this case. In the general Dirichlet-multinomial distribution there are *m *parameters, allowing far more than two (*α *and *β*) in the beta-binomial distribution. In situations where one is indeed concerned with multiple types of adverse events associated with the same exposure, expanding to the Dirichlet-multinomial distribution is a logical solution. Technical details of the multinomial model have been given by others [15-17].

### Test of overdispersion

Using the binomial model when the variability in the data exceeds what the binomial model can accommodate could result in an underestimation of the standard error of the pooled event rate and thus increase the chance of a Type I error. Ennis and Bi [5] described an experiment with 10,000 sets of simulated overdispersed binomial data where they found that the Type I error was 0.44 and not the false assumption of 0.05. It is precisely because the binomial model is unable to fit overdispersed binomial data that the application of the beta-binomial is necessary. So before one adopts the beta-binomial for the analysis of certain datasets, one must first examine whether the data are overdispersed to the extent that the beta-binomial model would be a better fit than the simple binomial model. There are several ways to examine overdispersion. We know that

(13) $$E(p_{i})=\mu =\frac{\alpha }{\alpha +\beta },V(p_{i})=\mu (1-\mu )\gamma$$

where *γ *= 1/(1 + *α *+ *β*). If we are able to estimate *γ*, we can test whether *γ *is zero. If it is close to zero, then there is no significant overdispersion, and the binomial model will adequately describe the data. This test, however, has been found to be less sensitive in detecting departure from the binomial model because boundary problems arise as we test whether a positive-valued parameter is greater than 0 (recall that *α *and *β *are positive parameters, and consequently so are *θ *and *γ*) [5].

As one would expect, a likelihood ratio test can also be used to test for overdispersion, but the same boundary problem applies [18,19]. The null hypothesis is that the underlying distribution is binomial while the alternative hypothesis is that the distribution is beta-binomial. The log-likelihood for the binomial model (interpreted to be pooling the data from all studies without weighting) is

(15) $$\ln L=\ln\left(\begin{array}{l} n\\ x \end{array}\right)+y\ln(p)+(n-x)\ln(1-p).$$

The likelihood ratio test is

(16) *χ*_1 _^2 ^= 2 (*L*_*BB *_- *L*_*B*_)

where *L*_*BB *_is the log-likelihood value for the beta-binomial model (9) and *L*_*B *_is log-likelihood value for the binomial model (15).

Although a solution for the boundary problem has been offered [20], there is no consensus on the optimal solution [21]. To avoid the boundary problem, we can use the alternative – Tarone's Z statistic [22] – to test for overdispersion. This has been shown to be more sensitive than the parameter test (e.g. test for *γ *being zero) and the log-likelihood ratio test [5]:

(14) $$Z=\frac{E-\sum_{i=1}^{k}n_{i}}{\sqrt{2\sum_{i=1}^{k}n_{i}(n_{i}-1)}}$$

where

$$\begin{array}{ccc} E=\sum_{i=1}^{k}\frac{{\left(x_{i}-n_{i}\hat{p}\right)}^{2}}{\hat{p}(1-\hat{p})} & \text{and} & \hat{p}=\sum_{i=1}^{k}\frac{x_{i}}{n_{k}}. \end{array}$$

This statistic *Z *has an asymptotic standard normal distribution under the null hypothesis of a binomial distribution. In short, we recommend caution in using the likelihood ratio test. It is better to combine it with Tarone's *Z *statistics. The *Z *statistics can also be used as a goodness-of-fit test. It has been shown to be superior to other goodness-of-fit measures [21]. We will be calculating Tarone's *Z *in our application example.

### The Bayesian Approach

In the preceding sections we describe the beta-binomial model within the frequentist framework of statistics. Interestingly, in the Bayesian statistics field, the beta-binomial model is commonly described in Bayesian statistics textbooks as an example [23,24]. Since Bayesian statistical methods are now increasingly used in clinical and public health research, we hereby briefly describe the derivation of the beta-binomial model in the Bayesian framework. Some have noted that the Bayesian approach can provide more accurate estimates for small samples [25,26].

Recall that the binomial distribution (in equation 1) is the following:

$$\text{Prob}(X=x|p)=\left(\begin{array}{l} n\\ x \end{array}\right)\;p^{x}{\left(1-p\right)}^{n-x}$$

Let the conjugate prior *π*(*p*|*α, β*) be a beta distribution (i.e., if *p *in equation 1 follows the beta distribution)

(17) $$\text{Beta}(p|\alpha ,\beta )=\frac{\Gamma (\alpha +\beta )}{\Gamma (\alpha )\Gamma (\beta )}p^{\alpha -1}{\left(1-p\right)}^{\beta -1}$$

where *Γ *is the gamma function. The beta priors are selected because they are very flexible on (0, 1) and can represent a wide range of prior beliefs. These are similar to the reasons for selecting the beta distribution in the frequentist framework. In addition, by starting with the beta distribution as the conjugate prior, we ensure that the posterior distribution is always a beta distribution, and thus mathematically tractable for estimating the parameters.

For notational convenience, let *μ = α/(α+β), M = α+β *(*i.e. M *= 1/*θ*), so that

$$\text{Beta}(p|\alpha ,\beta )=\frac{\Gamma (M)}{\Gamma (\mu M)\Gamma (M(1-\mu ))}p^{M\mu -1}{\left(1-p\right)}^{M(1-\mu )-1}$$

In short, we again have a two-stage model:

*X*_*i*_|*p*_*i*_~*Bin*(*n*_*i*_, *p*_*i*_)

*p*_*i*_~*Beta *(*μ, M*), *i.i.d*

In the Bayesian terminology, the beta prior distribution, when updated with binomial data, gives a beta posterior distribution. The Bayesian estimator can then be chosen as the mean, median, or the mode of this marginal posterior. In many situations, as long as the sample sizes are reasonably large (n = 50 or more), our previous methods of moment estimation and maximum likelihood are still preferred in the Bayesian framework for the estimations of mean and variance. There are other detailed mathematical equations involved in Bayesian estimation of the beta-binomial model for specific cases. Interested readers could consult Lee and Sabavala [25] as well as Lee and Lio [26].

## Results

We will illustrate the application of the beta-binomial method using an analysis that examined the adverse effects of oral anti-fungal agents. Oral anti-fungal agents, including terbinafine, itraconazole, and fluconazole, have become the treatment of choice for onychomycosis and dermatophytosis not responding to topical therapy. In order to study the safety profiles of these agents, we reviewed data from randomized and non-randomized controlled trials, case series, and cohort studies that enrolled patients having superficial dermatophytosis (tinea pedis, tinea mannus, tinea copora, and tinea cruris) or onychomycosis, aged 18 or above, receiving oral antifungal therapy for two or more weeks. One outcome of interest was the cumulative incidence of patients who withdrew from the study because of adverse reactions [7]. Data for 41 treatment arms of terbinafine from 37 studies (Table 1 and Appendix) are used as an example.

**Table 1:** Treatment arms of terbinafine included in pooled estimates

Treatment Arm*	Sample Size (No. of Patients – n_i_)	No. of Treatment Termination Due to Adverse Effect (x_i_)	Proportion of Treatment Termination (p_i _= x_i_/n_i_)
1	184	7	3.80%
2	65	1	1.54%
3	33	1	3.03%
4	151	4	2.65%
5	24	0	0.00%
6	30	0	0.00%
7	20	0	0.00%
8	22	0	0.00%
9	50	4	8.00%
10	50	5	10.00%
11	18	0	0.00%
12	26	0	0.00%
13	72	0	0.00%
14	30	1	3.33%
15	16	0	0.00%
16	26	2	7.69%
17	95	8	8.42%
18	95	3	3.16%
19	186	0	0.00%
20	146	11	7.53%
21	142	2	1.41%
22	124	8	6.45%
23	56	1	1.79%
24	12	0	0.00%
25	50	0	0.00%
26	88	3	3.41%
27	48	0	0.00%
28	75	4	5.33%
29	76	0	0.00%
30	56	1	1.79%
31	153	9	5.88%
32	68	1	1.47%
33	120	13	10.83%
34	44	0	0.00%
35	84	0	0.00%
36	21	0	0.00%
37	145	3	2.07%
38	83	10	12.05%
39	68	3	4.41%
40	30	3	10.00%
41	120	3	2.50%

*Referenced separately in appendix

Event rates from different studies varied from 0 % to 13.89%. We apply the beta-binomial model with the maximum likelihood method to estimate the pooled event rates using SAS and SAS macro BETABIN. From all the eligible studies, we combine the data and obtain the summary estimate of risks and its 95% confidence intervals (CI).

The ML estimates for parameters *μ *and *θ *are $\hat{\mu }$ = 0.0344 and $\hat{\theta }$ = 0.0278. The estimate of the covariance matrix for $\hat{\mu }$ and $\hat{\theta }$ is

$$Cov(\hat{\mu },\hat{\theta })=\left[\begin{array}{cc} 0.00004 & 0.00002\\ 0.00002 & 0.00013 \end{array}\right].$$

In Table 2, we present different estimations of a pooled proportion (event rates) using the binomial model and the beta-binomial model. Using the binomial model, we compute a binomial probability and variance as if all the data were from a single study with a sample size of over 3,000. The pooled estimate is 3.70%, 8% higher than the beta-binomial estimate of 3.44%. The standard error from the collapsed data is 0.34%, misleadingly smaller than that of the beta-binomial estimation of 0.59%.

**Table 2:** Estimation of proportion and tests of overdispersion

Methods	Estimate	Standard Error	Lower 95% CI	Upper 95% CI
Simple collapsed binomial	3.70%	0.34%	3.03%	4.34%
Beta-Binomial	3.44%	0.59%	2.28%	4.61%
Meta-analysis^1^	3.90%	0.61%	2.70%	5.09%

Test of Overdispersion				

	Estimate	Standard Error	Statistic	p-value
Alpha	1.24	0.52	z = 2.40	0.02
Beta	34.7	15.08	z = 2.30	0.02
Theta	2.78%	1.20%	z = 2.31	0.02
Gamma	2.71%	1.14%	z = 2.38	0.02
Likelihood Ratio^2^			X_1_^2 ^= 129.91	< 0.001
Tarone's Z			z = 7.95	< 0.001

**Figure 2:**
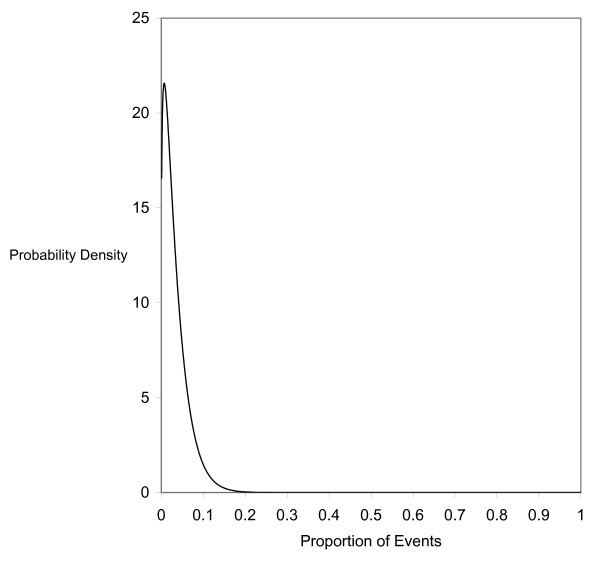
Beta distribution for the binomial proportions based on example.

The important issue naturally is the test of overdispersion since that is the basis for preferring the beta-binomial model in these situations. Results from different methods to evaluate overdispersion are presented in Table 2. As discussed in previous sections, *θ *and *γ *are indicators of overdispersion. They are significantly greater than zero in this case (p < 0.05), indicating the presence of overdispersion. We also conduct a likelihood-ratio test between the beta-binomial and the binomial, and again the test shows that there is significant overdispersion (p < 0.001). Finally, we calculate Tarone's *Z *statistic, and the result is consistent with other tests. It shows that the beta-binomial has better goodness-of-fit than the binomial (p < 0.001). The fit that the beta-binomial model gives for our example is also graphically presented in Figure 2.

As we have shown above, under the beta-binomial model the summary event rate is 3.44% with an estimated standard error of 0.59%. The *θ *is estimated to be 2.78% (Table 2), which gives an *α *estimate of 1.24 and a *β *estimate of 34.72. Once these parameters are estimated, we can use the estimated beta-binomial model to examine the probability of observing, for example, 105 or more adverse events in a new study of 1,000 subjects. Using equation 3, that probability is 5% under our estimated beta-binomial model.

## Discussion

Along with the development of drugs, vaccines, and medical products for unmet medical needs, more robust analytic methods are needed to quantify the risks associated with the use of these agents, so that regulators and clinicians can rigorously assess the risk-benefit profiles of medical products. While randomized controlled trials have been established as the gold standard for efficacy evaluation, comprehensive safety assessment requires a collection of different methods. As any single trial is rarely large enough to estimate precisely the probability of serious adverse events, large observational datasets or aggregations of clinical trial results are necessary. A recent high profile example [27] illustrated the need to combine results from multiple studies to unearth safety signals that may not be apparent in individual studies. Developing on prior work by Chuang-Stein [2], we provide a more comprehensive background of the beta-binomial model, a model that could have wider application in clinical and public health research. In order to show new developments in the beta-binomial field over the past decade, we explain and demonstrate that the beta-binomial method can be used for the combination of heterogeneous studies to estimate event rates.

Estimating the correct summary event rate based on heterogeneous binomial data is so far the main reason for adopting the beta-binomial distribution. Once this is accomplished, one might wish to examine whether specific attributes of the studies will have any meaningful impact. The beta-binomial model can incorporate these attributes into a regression model as covariates. For example, the main purpose of the study might be to evaluate the proportion of adverse events from all clinical trials involving drug A. Different studies might have different proportions of female subjects, and one may link the covariate, the proportion of female subjects, to the *α *parameter. In addition, different studies might include or exclude certain comorbid conditions. The comorbidity, defined as a binary variable, could also be included as a covariate. One can then evaluate the likelihood of the comorbidity increasing a specific side effect. As current meta-regression methods are mainly applied to comparative measures like relative risks, the advantage of the beta-binomial model is that it can assess the correlation between study attributes and absolute risks of events.

Traditional meta-analysis can also combine event rates from heterogeneous sources by using the DerSimonian and Laird method [28]. We applied this method to the same dataset and placed the summary rate in Table 2, with an estimate of 3.90% with a standard error of 0.51%. This is in good agreement with our estimation using the beta-binomial model. In medical product safety assessment, however, being able to derive a clear probability distribution offers advantages that traditional meta-analysis cannot, because the distributions allow the computation of absolute risks or probabilities involved in decision analysis. In the Bayesian framework, the beta-binomial model also enables better incorporation of prior knowledge and its associated uncertainty. In other words, even though traditional meta-analysis can also combine event rates, the adoption of the beta-binomial model can serve multiple purposes.

## Conclusion

In the process of pooling event rates from multiple studies, one must consider the existence of overdispersion and the adequacy of the binomial model. In the example that we have presented, we estimated the pooled proportion of adverse events using the beta-binomial model. While we mainly discussed the application in safety assessment, the same method can be applied to assessment of efficacy of treatment response [29].

## Appendix

Studies Included in Table 1

1. Alpsoy E, Yilmaz E, Basaran E. Intermittent therapy with terbinafine for dermatophyte toe-onychomycosis: a new approach. J Dermatol. 1996:23:259–262.

2. Arca E, Taştan HB, Akar A, Kurumlu Z, Gür AR. An open, randomized, comparative study of oral fluconazole, itraconazole and terbinafine therapy in onychomycosis. J Dermatolog Treat. 2002:13:3–9.

3. Arenas R, Dominguez-Cherit J, Fernandez LM. Open randomized comparison of itraconazole versus terbinafine in onychomycosis. Int J Dermatol. 1995:34:138–143.

4. Avner S, Nir N, Henri T. Combination of oral terbinafine and topical ciclopirox compared to oral terbinafine for the treatment of onychomycosis. J Dermatolog Treat. 2005;16:327–330.

5. Baldari U, Righini MG, Raccagni AA, et al. Comparative double blind, double dummy study on the efficacy and safety of fluconazole 100 mg/day versus terbinafine 250 mg/day in the treatment of dermatomycoses. G Ital Dermatol Venereol. 2000;135:229–235.

6. Baran R, Belaich S, Beylot C, et al. Comparative multicentre doubleblind study of terbinafine (250 mg per day) versus griseofulvin (1 g per day) in the treatment of dermatophyte onychomycosis. J Dermatolog Treat. 1997;8:93–97.

7. Baran R, Feuilhade M, Combernale P, et al. A randomized trial of amorolfine 5% solution nail lacquer combined with oral terbinafine compared with terbinafine alone in the treatment of dermatophytic toenail onychomycoses affecting the matrix region. Br J Dermatol. 2000;142:1177–1183.

8. Brautigam M, Nolting S, Schopf RE, Weidinger G. Randomised double blind comparison of terbinafine and itraconazole for treatment of toenail tinea infection. Seventh Lamisil German Onychomycosis Study Group. BMJ. 1995;311:919–922.

9. De Backer M, De Vroey C, Lesaffre E, et al. Twelve weeks of continuous oral therapy for toenail onychomycosis caused by dermatophytes: a double-blind comparative trial of terbinafine 250 mg/day versus itraconazole 200 mg/day. J Am Acad Dermatol. 1998;38 (5 Pt 3):S57–S63.

10. De Keyser P, De Backer M, Massart DL, Westelinck KJ. Two-week oral treatment of tinea pedis, comparing terbinafine (250 mg/day) with itraconazole (100 mg/day): a double-blind, multicentre study. Br J Dermatol. 1994;130(Suppl 43):22–25.

11. Degreef H, del Palacio A, Mygind S, et al. Randomized double-blind comparison of short-term itraconazole and terbinafine therapy for toenail onychomycosis. Acta Derm Venereol. 1999;79:221–223.

12. del Palacio Hernandez A, Lopez Gomez S, Gonzalez Lastra F, et al. A comparative double-blind study of terbinafine (Lamisil) and griseofulvin in tinea corporis and tinea cruris. Clin Exp Dermatol. 1990;15:210–216.

13. Drake LA, Shear NH, Arlette JP, et al. Oral terbinafine in the treatment of toenail onychomycosis: North American multicenter trial. J Am Acad Dermatol. 1997;37(5 Pt 1):740–745.

14. Evans EG, Sigurgeirsson B. Double blind, randomised study of continuous terbinafine compared with intermittent itraconazole in treatment of toenail onychomycosis. The LION Study Group. BMJ. 1999;318:1031–1035.

15. Faergemann J, Anderson C, Hersle K, et al. Double-blind, paralle-lgroup comparison of terbinafine and griseofulvin in the treatment of toenail onychomycosis. J Am Acad Dermatol. 1995;32(5 Pt 1):750–753.

16. Goodfield MJ, Andrew L, Evans EG. Short term treatment of dermatophyte onychomycosis with terbinafine. BMJ. 1992;304:1151–1154.

17. Goodfield MJ, Rowell NR, Forster RA, et al. Treatment of dermatophyte infection of the finger- and toe-nails with terbinafine (SF86-327, Lamisil), an orally active fungicidal agent. Br J Dermatol. 1989;121:753–757.

18. Gupta AK, Gregurek-Novak T. Efficacy of itraconazole, terbinafine, fluconazole, griseofulvin and ketoconazole in the treatment of Scopulariopsis brevicaulis causing onychomycosis of the toes. Dermatology. 2001;202:235–238.

19. Gupta AK, Konnikov N, Lynde CW, et al. Single-blind, randomized, prospective study on terbinafine and itraconazole for treatment of dermatophyte toenail onychomycosis in the elderly. J Am Acad Dermatol 2001; 44: 479–484.

20. Haneke E, Tausch I, Brautigam M, et al. Short-duration treatment of fingernail dermatophytosis: a randomized, double-blind study with terbinafine and griseofulvin. LAGOS III Study Group. J Am Acad Dermatol. 1995;32:72–77.

21. Havu V, Heikkila H, Kuokkanen K, et al. A double-blind, randomized study to compare the efficacy and safety of terbinafine (Lamisil) with fluconazole (Diflucan) in the treatment of onychomycosis. Br J Dermatol. 2000;142:97–102.

22. Hay RJ, McGregor JM, Wuite J, et al. A comparison of 2 weeks of terbinafine 250 mg/day with 4 weeks of itraconazole 100 mg/day in plantar-type tinea pedis. Br J Dermatol. 1995;132:604–608.

23. Hofmann H, Brautigam M, Weidinger G, Zaun H. Treatment of toenail onychomycosis. A randomized, double-blind study with terbinafine and griseofulvin. LAGOS II Study Group. Arch Dermatol. 1995;131:919–922.

24. Honeyman JF, Talarico FS, Arruda LHF, et al. Itraconazole versus terbinafine (LAMISIL(registered trademark)): which is better for the treatment of onychomycosis? J Eur Acad Dermatol Venereol. 1997; 9:215–221.

25. Kim JH, Yoon KB. Single-blind randomized study of terbinafine vs itraconazole in tinea pedis (two weeks vs four weeks). Terbinafine in the treatment of superficial fungal infections, edited by S. Shuster and M. H. Jafary, 1993; p17-20 Royal Society of Medicine Services International Congress and Symposium Series No. 205, published by Royal Society of Medicine Services Limited.

26. Savin R. Successful treatment of chronic tinea pedis (moccasin type) with terbinafine (Lamisil). Clin Exp Dermatol. 1989;14:116–119.

27. Savin RC, Zaias N. Treatment of chronic moccasin-type tinea pedis with terbinafine: a double-blind, placebo-controlled trial. J Am Acad Dermatol. 1990;23(4 Pt 2):804–807.

28. Svejgaard EL, Brandrup F, Kragballe K, et al. Oral terbinafine in toenail dermatophytosis. A double-blind, placebo-controlled multicenter study with 12 months' follow-up. Acta Derm Venereol. 1997; 77:66–69.

29. Tausch I, Brautigam M, Weidinger G, Jones TC. Evaluation of 6 weeks treatment of terbinafine in tinea unguium in a double-blind trial comparing 6 and 12 weeks therapy. The Lagos V Study Group. Br J Dermatol. 1997;136:737–742.

30. Tausch I, Decroix J, Gwiezdzinski Z, et al. Short-term itraconazole versus terbinafine in the treatment of tinea pedis or manus. Int J Dermatol. 1998;37:140–142.

31. Tosti A, Piraccini BM, Stinchi C, et al. Treatment of dermatophyte nail infections: an open randomized study comparing intermittent terbinafine therapy with continuous terbinafine treatment and intermittent itraconazole therapy. J Am Acad Dermatol. 1996;34:595–600.

32. van der Schroeff JG, Cirkel PK, Crijns MB, et al. A randomized treatment duration-finding study of terbinafine in onychomycosis. Br J Dermatol. 1992;126(Suppl 39):36–39.

33. Voravutinon V. Oral treatment of tinea corporis and tinea cruris with terbinafine and griseofulvin: a randomized double blind comparative study. J Med Assoc Thai. 1993;76:388–393.

34. Warshaw, E.M., D.D. Fett, H.E. Bloomfield, J.P. Grill, D.B. Nelson, V. Quintero, S.M. Carver, G.R. Zielke and F.A. Lederle. Pulse versus continuous terbinafine for onychomycosis: a randomized, double-blind, controlled trial. J Am Acad Dermatol. 2005;53:578–584.

35. Watson A, Marley J, Ellis D, Williams T. Terbinafine in onychomycosis of the toenail: a novel treatment protocol. J Am Acad Dermatol. 1995;33(5 Pt 1):775–779.

36. Widyanto BU, Kuswadji KB. A randomized, double blind comparative study of terbinafine vs griseofulvin in tinea pedis. Terbinafine in the treatment of superficial fungal infections, edited by S. Shuster and M. H. Jafary, 1993, p21-24; Royal Society of Medicine Services International Congress and Symposium Series No. 205, published by Royal Society of Medicine Services Limited.

37. Won YH, Kim SJ, Lee HW, Chun IK. Clinical comparative study of terbinafine and itraconazole in the treatment of tinea pedis. Terbinafine in the treatment of superficial fungal infections, edited by S. Shuster and M. H. Jafary, 1993, p7-10; Royal Society of Medicine Services International Congress and Symposium Series No. 205, published by Royal Society of Medicine Services Limited.

## Competing interests

Both authors work part-time for for-profit companies (YYX for EpiPatterns and KAC for i3 Drug Safety). KAC received support from the Harvard Pharmacoepidemiology program, which has received unrestricted funds from pharmaceutical companies.

## Authors' contributions

YYX and KAC conceived of the study. YYX performed the statistical analysis and wrote the manuscript. KAC participated in the analysis of the study and the writing of the manuscript. Both authors read and approved the final manuscript.

## Pre-publication history

The pre-publication history for this paper can be accessed here:


